# Dynamic Editome of Zebrafish under Aminoglycosides Treatment and Its Potential Involvement in Ototoxicity

**DOI:** 10.3389/fphar.2017.00854

**Published:** 2017-11-22

**Authors:** Sijia Yan, Yulan Lu, Lin He, Xinzhi Zhao, Lihua Wu, Huizhong Zhu, Menglin Jiang, Yu Su, Wei Cao, Weidong Tian, Qinghe Xing

**Affiliations:** ^1^Institutes of Biomedical Sciences and Children's Hospital, Fudan University, Shanghai, China; ^2^Children's Hospital, Fudan University, Shanghai, China; ^3^Bio-X Institutes, Key Laboratory for the Genetics of Developmental and Neuropsychiatric Disorders (Ministry of Education), Shanghai Jiao Tong University, Shanghai, China; ^4^Zhengzhou People's Hospital, Zhengzhou, China; ^5^Zhengzhou Central Hospital, Zhengzhou University, Zhengzhou, China; ^6^Department of Biostatistics and Computational Biology, School of Life Science, Fudan University, Shanghai, China

**Keywords:** zebrafish, RNA-editing, RNA-Seq, aminoglycosides (AG), ototoxicity

## Abstract

RNA editing is an important co- and post-transcriptional event that generates RNA and protein diversity. Aminoglycosides are a group of bactericidal antibiotics and a mainstay of antimicrobial therapy for several life-threatening infections. However, aminoglycosides can induce ototoxicity, resulting in damage to the organs responsible for hearing and balance. At low concentrations, aminoglycosides can bind to many RNA sequences and critically influence RNA editing. We used a bioinformatics approach to investigate the effect of aminoglycosides on global mRNA editing events to gain insight into the interactions between mRNA editing and aminoglycoside ototoxicity. We identified 6,850 mRNA editing sites in protein coding genes in embryonic zebrafish, and in about 10% of these, the degree of RNA editing changed more than 15% under aminoglycosides treatment. Twelve ear-development or ototoxicity related genes, including *plekhm1, fgfr1a, sox9a*, and *calrl2*, exhibited remarkable changes in mRNA editing levels in zebrafish treated with aminoglycosides. Our results indicate that aminoglycosides may have a widespread and complicated influence on the progress of mRNA editing and expression. Furthermore, these results highlight the potential importance of mRNA editing in the pathogenesis and etiology of aminoglycoside-induced ototoxicity.

## Introduction

RNA editing is a co- and post-transcriptional mechanism of introducing changes into RNA sequences encoded by the genomic blueprint. RNA editing finely regulates gene function including splicing, localization, translation, and transcript stability. The most common type of RNA editing in metazoans is the conversion of adenosine to inosine, which is translated as guanosine [A-to-I (G)]. This change is carried out by adenosine deaminases that act on RNA (ADARs) proteins, a family of double stranded RNA (dsRNA) binding enzymes. Consequently, these modifications can alter codon identity and increase genetic diversity (Nishikura, 2010). In human, A-to-I (G) editing sites are mostly located in Alu repeats, and are essential for the normal physiology of cells (Wang et al., 2013; Porath et al., 2014). Moreover, this mechanism is not static, and shows continuous dynamic change in different tissues and development stages to fine-tune and optimize biological pathways (Mehler and Mattick, 2007; Hwang et al., 2016; Qiu et al., 2016).

RNA editing level basically maintained within normal range is an essential mechanism to maintain normal physiological function. The deregulation of RNA editing may contribute to neurological diseases such as epilepsy, depression, schizophrenia, autism, fragile-X syndrome, Alzheimer's disease, Huntington's disease, and amyotrophic lateral sclerosis (Akbarian et al., 1995; Kawahara et al., 2004; Maas et al., 2006; Gallo et al., 2017). The editing sites in *AZIN1* play an important role in hepatocellular carcinoma tumorigenesis, and two editing sites in *COG3* and *SRP9* have been reported in a breast cancer study (Shah et al., 2009; Chen et al., 2013). Therefore, RNA editing deficiencies or hyperactivity may be associated with additional, as yet undiscovered, pathological mechanisms.

ADARs recognize dsRNA substrates characterized by loops and bulges (Wong et al., 2001), and ADAR2 preferentially binds imperfect RNA fold-back structures (Klaue et al., 2003). Numerous results suggest that RNA is a major biological target of aminoglycosides (AG; Vicens and Westhof, 2003). AG are clinically important antibiotics despite their unwanted side effects of ototoxicity and nephrotoxicity (Rizzi and Hirose, 2007). AG exert their antibiotic effects through binding to the A site of bacterial 16S rRNA (Kaul and Pilch, 2002). Moreover, positively charged amino groups facilitate AG docking to negatively charged pockets in RNA folds (Hermann and Westhof, 1999). Direct observation of AG–RNA interactions shows that neomycin B generally binds to regular A-form RNA or hairpin loops (Hendrix et al., 1997). Therefore, neomycin B has been used as a positive control in the inhibition of ADAR2-catalyzed editing of certain substrates (Schirle et al., 2010). Given that dsRNA is the substrate of RNA editing and AG binding, we hypothesized that AG may interfere with, or change, normal RNA editing.

Zebrafish embryos possess transparent bodies, making it easy to observe structural changes. Moreover, the lateral line hair cells of zebrafish share essential properties with human inner ear hair cells, and thus they are often used as a model for studying drug toxicity (Kari et al., 2007). Earlier studies only identified a handful of RNA editing sites in zebrafish (Sie and Maas, 2009; Pozo and Hoopengardner, 2012; Li et al., 2014). A recent study identified more than 300 thousands of clustered RNA editing sites in the zebrafish covering eight different developmental stages, in which 5,460 editing sites were detected in the gene coding sequences (Shamay-Ramot et al., 2015). However, the global extent of editome changes in gene body of zebrafish embryos after treatment with AG has not yet been investigated.

In principle, RNA editing sites can be inferred based on sequence differences between RNA (or cDNA) and the genomic DNA from which it is expressed. Here, we focused on mRNA editing sites because mRNA plays a critical role in the central dogma of molecular biology. We firstly identified post-transcriptional editing events in control or AG treated zebrafish using both mRNA-seq and DNA-seq. DNA-seq data was used to filter the DNA variants. mRNA-seq data were analyzed by a novel bioinformatic pipeline aimed at detecting hyper RNA editing sites (Zhang et al., 2017). Our improved approach revealed 6,850 mRNA editing sites in gene body regions, including coding sequences (CDS), untranslated regions (UTR), and introns. Our results provide a survey of the variation in mRNA editing rates after treatment with ototoxic drugs, such as AG. As the precise mechanism of AG-induced ototoxicity has not yet been fully elucidated, determining the global and dynamic aspects of the editome and transcriptome under AG treatment could inform a new area of research and provide new insights into the epitranscriptional regulation of sequence diversity.

## Materials and methods

### Animals

Zebrafish (AB line, standard length: 2.7–3.5 cm) were raised in a recirculating water system according to standard protocols (Westerfield, 2000). Aquaria-system water was dosed to a salinity of 500 μF with artificial ocean salt mix and buffered to pH 7.2 with NaHCO_3_. After the group mating of four male and four female adult zebrafish (aged 6 months post-fertilization), embryos were collected and raised at 28.5°C in embryo medium (EM) at a density of 35–40 embryos per 10-cm diameter Petri dish. EM was prepared as previously reported (Coffin et al., 2009). Embryos were staged by hours post-fertilization (hpf) and days post-fertilization (dpf) as described previously (Kimmel et al., 1995). This study was carried out in accordance with the recommendations of the Fudan University Institutional Animal Care and Use Committee's guidelines (20120302-065). The protocol was approved by the Fudan University Institutional Animal Care and Use Committee.

### Aminoglycoside treatment

Neomycin (5 g stock) or gentamicin (5 g stock; Sangon Biotech) was diluted in EM to final concentrations of 200 μM, or 25 and 50 μM, respectively. AG exposure paradigms were based on prior observations of the zebrafish lateral line (Owens et al., 2009; Stengel et al., 2017).

Healthy staged embryos were incubated from the 50% epiboly of embryonic shield stage (5.25 hpf) in neomycin or gentamicin to the long-pec stage during the hatching period (2 dpf). The medium was changed every 24 h. There was no statistical difference in embryo mortality between the exposed groups and control groups during this period. The 2 dpf zebrafish embryos were rinsed with EM several times and transferred to EM for further observation. The control groups were raised in EM the entire time, and the EM was changed every 24 h. Hair cell damage was examined in zebrafish embryos at 2 and 4 dpf.

Hair cells were pre-labeled with the mechanotransduction marker FM1-43 FX (3 μM in standard aquaria system water; Invitrogen Molecular Probes) for 45–60 s. The embryos were then quickly rinsed three times with EM. Using this procedure, FM1-43 FX is restricted to hair cells in neuromasts (Seiler and Nicolson, 1999). Hair cell survival was denoted by FM1-43 FX-positive cytoplasm surrounding the nucleus and an intact cell morphology.

Embryos were anesthetized with 0.001% MS-222 (Sigma). Zebrafish observations and image capture were conducted on a Zeiss Discovery V.20 microscope with a GFP filter set and an AxioCamHRc camera (Carl Zeiss), to visualize FM1-43 FX. Image stack projections or single image slices were exported from Slidebook software v. 4 (Olympus).

### RNA isolation and deep sequencing

Total RNA for each sample was collected from 30 to 35 embryos at 2 dpf. Total RNA was isolated using Trizol reagent (Life Technologies). Agilent 2100 Bioanalyzer was used to characterize *in vitro* RNA transcripts for quality. The RNA integrity numbers (Rin) of all samples were higher than 8.0. The poly-A-containing mRNA molecules were purified using poly-T oligo-attached magnetic beads using two rounds of purification. A SuperScript Double-Stranded cDNA Synthesis kit (Invitrogen) was used to synthesize the double-stranded cDNA. Further library preparation was performed using TruSeq™ RNA Sample Preparation Kit (Illumina, cat# FC-122-1001). Libraries were sequenced as SR 2 × 100 bp using Illumina Hiseq2000 according to the manufacturer's instructions. We removed adaptor, low-complexity, and low-quality sequences in the raw reads. The remaining clean reads were used for further analyses.

### DNA sequencing of zebrafish

Genomic DNA was isolated from the whole bodies of the four male and four female adult zebrafish from the mating pool using the DNeasy Blood & Tissue Kit (Qiagen) according to the manufacturer's instructions. Equal amounts of total DNA from each zebrafish were pooled into a single sample. About 5 μg genomic DNA was sheared into fragments of 200–250 bp using a Covaris focused acoustic sonicator (Covaris, MA, USA). After size selection of fragments by 2% agarose gel electrophoresis, we constructed paired-end libraries with the NEXTflex DNA Sequencing Kit (BIOO Scientific, TX, USA) and Illumina adaptors using 1 μg of sheared input DNA. All libraries were sequenced on the Illumina Hiseq 2500 platform (Illumina Inc., CA, USA). After filtering out adaptor sequences, low-quality reads, and duplicate reads, a total of 54,802 Gb of data was retained for assembly.

### Variant calling

The first 10 nts of paired-end reads were trimmed and filtered using a sequencing quality score cut-off of 30 for each RNA-seq dataset. Filtered reads were then aligned against the zebrafish reference genome (UCSC danRer7) with the Burrows-Wheeler Aligner program (BWA, version 0.7.12) using the default parameters (Li and Durbin, 2009). For each DNA-seq dataset, the first 10 nts of paired-end reads were also trimmed before being aligned against the reference genome with BWA. After mapping, the Samtools mpileup function was used to remove PCR duplicates, sort the alignment file, and call variants using default parameters for both RNA-seq and DNA-seq datasets (Li et al., 2009).

To improve the efficiency and accuracy of the discovery of RNA editing sites, we applied data quality filtration criteria during the three major steps of the analysis. Firstly, we took variant positions in the RNA into consideration if they varied from the reference genome and met our requirements in terms of the number, frequency, and quality of bases. We specifically required that the read coverage was at least 10, the mapping quality score of covered reads was at least 20, and there were at least two reads to support the alternative allele (Shamay-Ramot et al., 2015). Secondly, all homozygous and heterozygous DNA variants identified from DNA-seq data were filtered. Variants located within intergenic regions, polymers longer than 5 nts, or simple repeat regions, as well as known zebrafish SNPs in dbSNP (database version 135; http://www.ncbi.nlm.nih.gov/SNP/) and Ensembl variants annotated in Zv9 were also filtered. Thirdly, to further reduce false positives and identify RNA editing variants, we used the RNA-editing Calling process from a recently published method called SPRINT, which uses the editing type and cluster information of potential RNA editing sites to further enhance the calling power (Zhang et al., 2017).

### Recalling and comparing the degree of editing

To demonstrate differences in RNA editing among various treatments, all candidate RNA editing sites were combined as a candidate group, and then the sequencing statuses of these sites were re-evaluated with Samtools. For each different treatment, we calculated the ratio of edited sites for each replicated sample and determined the mean value of the treatment group. The degree of editing for a given site was calculated as the ratio of reads supporting the edited base to the total number of reads covering the site.

### Validation of sites with PCR and sanger sequencing and sequence logo generation

PCR amplification of gDNA and cDNA was carried out with the GeneAmp® PCR System 9700 (Thermo Fisher Scientific, MA, USA). The PCR protocol was as follows: 95°C for 4 min; 30 cycles of 94°C for 30 s, 57°C for 30 s, and 72°C for 30 s; 72°C for 10 min; followed by storage at 4°C as necessary. Direct sequencing was performed using an ABI 3730 DNA Analyzer (Applied Biosystems, MA, USA). The primers used for Sanger sequencing validation of called sites were listed in Supplementary Table 10. Sequence logos were generated with the program Two sample logo (Vacic et al., 2006).

### Gene expression analysis

Tophat v2.1.0 was used with default parameters to generate acceptable alignments for Cufflinks, in which the RNA-seq paired-end reads were aligned against the reference genome, UCSC danRer7 (Trapnell et al., 2012). Annotated gene expression was evaluated in FPKM (fragments per kilobase per million mapped fragments) using Cufflinks from RNA-seq data. The formula to calculate FPKM was as follows: FPKM = (number of mapping fragments) × 10^3^ × 10^6^/ [(length of transcript) × (number of total fragments)]. The method of normalizing expression data for comparison included log transformation and zero-mean normalization. Pathway analysis of genes with mRNA editing sites in zebrafish was performed using PANTHER v.10 (Mi et al., 2016).

### Correlation analysis of expression and editing

The correlation between gene expression and editing was calculated using R software, version 3.1.2 (The R Foundation for Statistical Computing, Vienna, Austria; R Development Core Team, 2013).

### Predicting MicroRNA (miRNA) binding sites

For a given candidate RNA editing site, we used MIRANDA (Enright et al., 2003) to predict potential miRNA binding sites in both the genomic sequence and mRNA sequence after editing. Sequence windows of 50 bp around each editing site were provided to the prediction algorithm.

## Results

### Establishing a model of AG-induced ototoxicity

**Figure 1:**
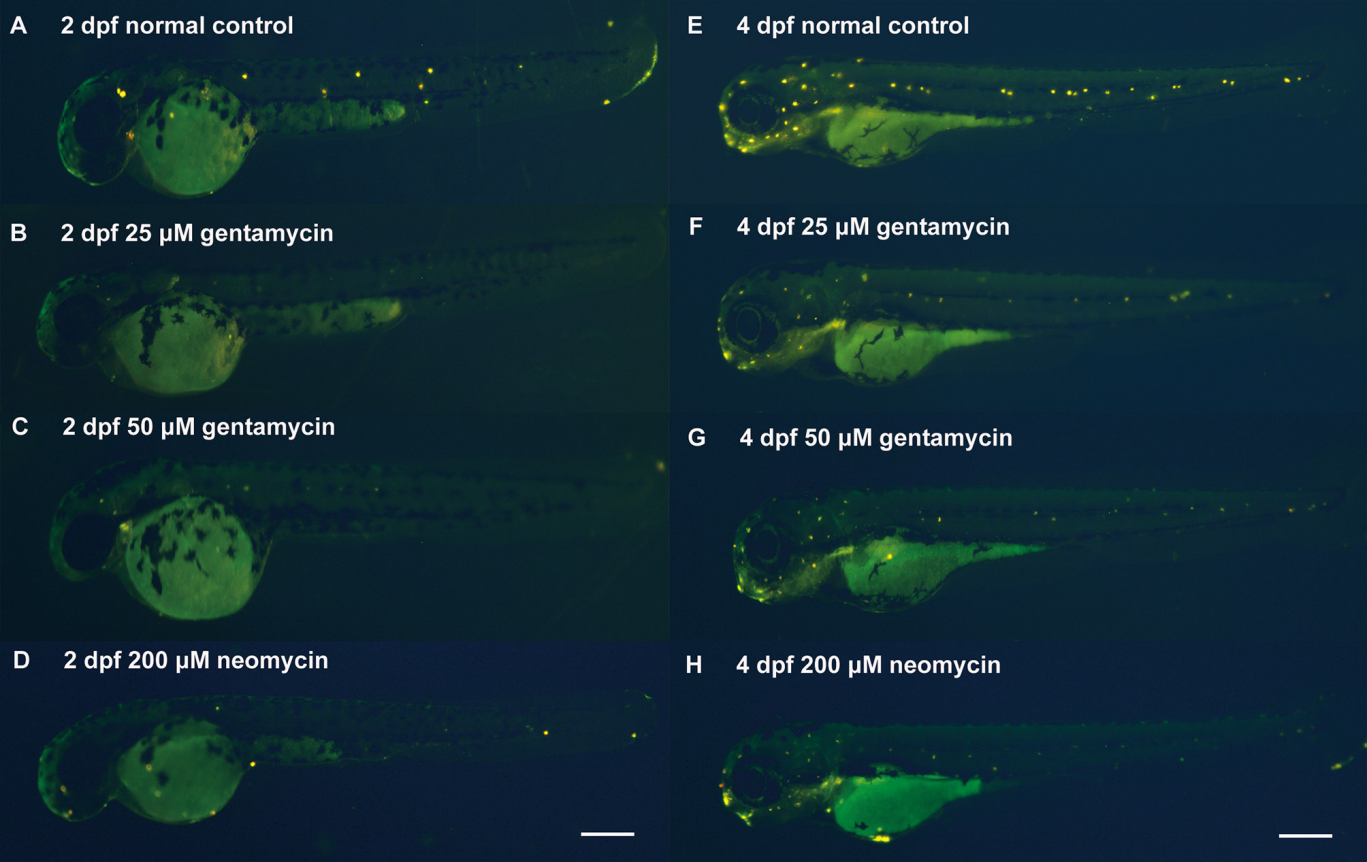
The zebrafish model of AG induced ototoxicity. **(A–F**, **E–H)** Reduced numbers of neuromast hair cells in AG treated fish labeled by FM1-43 FX at 2 or 4 dpf, respectively. The reduction in hair cells was statistically different in the control & treated groups.

To establish a model of AG-induced ototoxicity, embryonic zebrafish were incubated in medium containing various concentrations of AG from the beginning of zebrafish ear development at the 50% epiboly/shield stage to 2 dpf, when the statoacoustic (VIIIth) ganglion becomes a separate section (Haddon and Lewis, 1996; Whitfield et al., 2002). Then, AG-treated zebrafish embryos were cleansed with and transferred to normal EM until 4 dpf, when the mechanoelectrical transducer channels attained maturity (Santos et al., 2006). Analysis of FM1-43 FX hair cell labeling at 2 and 4 dpf revealed that hair cells had differentiated into neuromasts in the head and along the posterior lateral line in the control zebrafish (Kimmel et al., 1995). However, few fluorescently labeled hair cell bundles were observed in the head and lateral line neuromasts of 2 and 4 dpf zebrafish embryos treated with AG (Figures 1A–H). The reduction in hair cells in the lateral line labeled with FM1-43X indicates the failure of hair cells to mature or to normally function under AG treatment.

### DNA and mRNA sequencing of zebrafish samples

To identify mRNA editing sites, we first sequenced mRNA from the 2 dpf embryos of the AB zebrafish line. We sequenced RNA from a total of nine AG treatment and control groups. Then, we sequenced DNA from adult zebrafish that represented the parental generation of the embryos. A total of 367.49 million mRNA sequencing reads and 219.2 million DNA sequencing reads were obtained. An average of 40.8 million reads or 4.0 Gb of mRNA-Seq data were generated per mRNA sample with a read length of 100 bp (paired-ends) and expected insertion size of 200 bp. The alignment rate against the reference genome (see section Materials and Methods) ranged from 94.04% (sample Genta_50_rep2) to 95.78% (sample Genta_50_rep1), with average mapping rates of 95.06 and 94.83% for control and drug-treated samples, respectively (Supplementary Table 1).

### RNA editing calling in control and AG-treated samples

We identified an average of 6,973 edited RNA sites in gene body regions per RNA sample, with the number of edited sites ranging from 6,859 to 7,019. Exposure to 25 μM gentamycin resulted in hair cell loss similar to that of 50 μM gentamycin; therefore, we combined the data obtained from these samples in subsequent analyses. Finally, we identified 6,850 overlapping mRNA editing sites and calculated the level of editing in control, gentamycin, and neomycin-treated samples (Supplementary Table 2). Of the edited sites, 5,662 consisted of canonical, A-to-G or T-to-C editing. Two types of transitions, G-to-A and C-to-T, accounted for 77.4% of the non-canonical events observed.

We verified a subset of the edited RNA sites using PCR amplification and Sanger sequencing of DNA and mRNA (reverse-transcribed to cDNA). We examined 182 editing sites across 17 genes, including 13 G-to-A and C-to-T editing events. The validation results yielded a false-discovery rate of 5.33% (9/169) for A-to-G or T-to-C sites identified in our analysis, which was lower than the false-discovery rate for canonical sites in the previous study (Peng et al., 2012). The false-discovery rate for the non-canonical type was ~84.6% (11/13; Supplementary Table 3). The validation of editing events in the *sp4* and *si*:*ch211-114n24.7* genes is shown as representative examples (Supplementary Image 1).

### Characterization of mRNA editing sites

We identified 6,850 mRNA editing sites distributed over 762 genes. About 80% of these sites were A-to-G substitutions. The master list of editing sites contains 5,590 exonic sites, 1,243 intronic sites, and 17 sites located in regions with conflicting database annotations. Among exonic sites, the UTRs, especially the 3′ UTR, contained the greatest percentage of both A-to-G and non-A-to-G variants. CDS regions contained a total of 479 edited sites, of which 35% led to amino acid changes. Furthermore, there were significantly more non-A-to-G sites than A-to-G sites in CDS regions (*p* < 2.2 × 10^−16^). Among the 762 genes with editing sites identified in this study, 299 genes were reported to contain editing sites in previous studies of the human transcriptome (Peng et al., 2012; Ramaswami et al., 2013; Qiu et al., 2016), 54 genes had been described as edited in the mouse transcriptome (Danecek et al., 2012; Gu et al., 2012), and 325 genes had been described as edited in the zebrafish transcriptome (Shamay-Ramot et al., 2015). There were 279 genes which had not been described as edited before.

We also found that the RNA editing sites were clustered, with an average of nine editing sites per gene. The extent of A-to-G site clustering in our data set, with 60.06% of sites arranged in clusters of ≥3 sites within 100 bp, is lower than what is found in the DARNED database (85.02%) but is higher than that observed in another deep-sequencing data set acquired by Peng et al. (30.89%) (Kiran and Baranov, 2010; Peng et al., 2012). A large number of transcripts presented multiple editing sites (204 genes with ≥10 sites each), such as *sp4, slc22a31, plekhm1, adrbk2, pkn1*, and *samhd1*, which were validated with PCR and Sanger sequencing.

Pathway analysis of genes with mRNA editing sites in zebrafish was performed using PANTHER v.10 (Mi et al., 2016). mRNA editing sites were significantly enriched (*p* < 0.05) in the Huntington disease, semaphorin-mediated axon guidance, angiogenesis, pyrimidine metabolism, cytoskeletal regulation by Rho GTPase, and Alzheimer disease-presenilin and FGF signaling pathways (Supplementary Table 4). Moreover, *plch1* and *snap29*, part of the 5HT2-type receptor-mediated signaling pathway, also contained several editing sites. These findings indicate that RNA editing participates in the maintenance and regulation of zebrafish development, and particularly in neural development.

### Comparison of the degree of editing between AG-treated and control samples

RNA editing can affect anywhere from 0 to 100% of an RNA population and leads to a mixed RNA population. To explore the influence of AG on the mRNA editome, we investigated differences in the degree of editing between AG-treated and control samples (**Figure 3A**). Editing sites with more than a 15% change in the degree of editing in both AG treatments were included in the high variation group and used for further analysis. Notably, in the high variation group, 687 sites spanning 333 genes accounted for 43% of all detectable RNA-edited genes (Supplementary Table 5). Of these RNA editing sites, 462 and 225 showed a lower and higher degree of editing, respectively, in AG-treated samples than in control samples. In the high variation group, canonical variants (A-to-G or T-to-C) accounted for 75.8% of all RNA edits. The average value of the editing degree variation was about 26% among down-regulated edits and 27% among up-regulated edits. A total of 21 editing events across 16 genes were likely to change the encoded amino acids, and 57% of these changes were non- canonical variants. Pathway analysis showed that the 333 genes in the high variation group were significantly enriched (*p* < 0.05) in axon guidance mediated by netrin and in the FGF and Ras signaling pathways (Supplementary Table 6).

**Figure 2:**
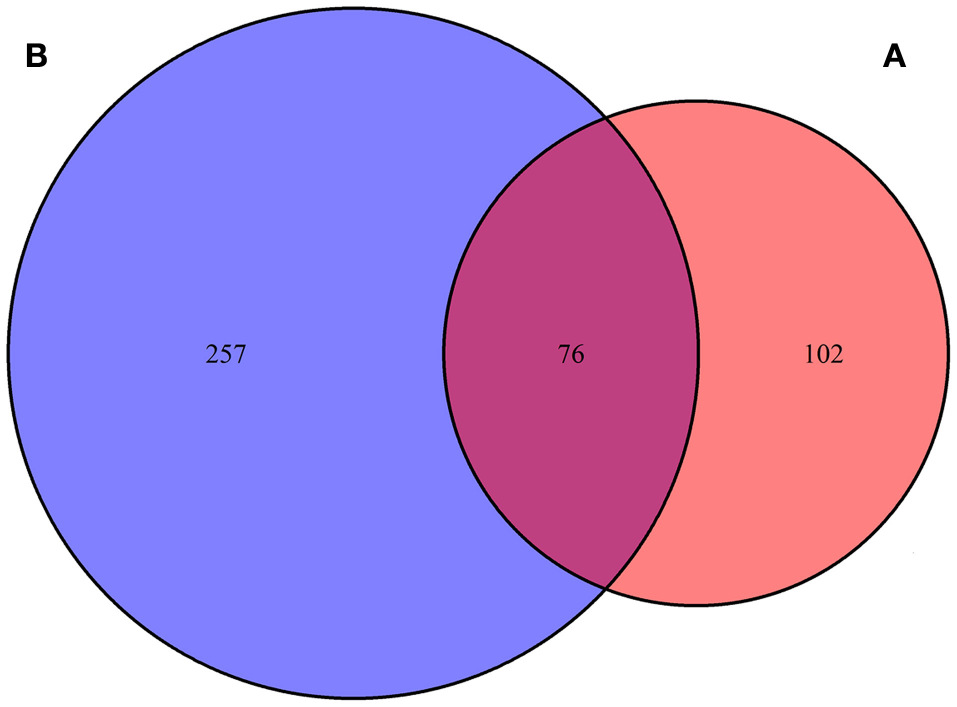
Venn-diagram highlighting percentage of genes with editing sites changed more than 15% in lateral line cell enriched genes. **(A)** One hundred and seventy-eight genes with editing events were significantly enriched in lateral line cells or lateral line placode cells, **(B)** 333 genes contained editing events with editing variations more than 15% between control and AG treatment samples.

A total of 178 genes with editing events were significantly enriched in lateral line cells or lateral line placode cells, with 76 genes containing editing events with editing variations of more than 15% between control and AG-treated samples (Jiang et al., 2014; Steiner et al., 2014; Figure 2). Moreover, 24 editing sites were identified as having more than a 15% change in the level of editing following AG treatment in 12 genes potentially related to ototoxicity, including *plekhm1, fgfr1a, sox9a*, and *calrl2* (Karasawa et al., 2011; Girotto et al., 2013; Stamatiou and Stankovic, 2013; Azaiez et al., 2014; Table 1). Editing events in the deafness-related genes *bdp1* and *alms1* led to amino acid changes, which may be one of the potential pathways of AG-induced ototoxicity. Other important genes with editing events with more than 15% variation that may change the amino acid sequence are listed in Table 2. Furthermore, editing variation in the mitochondrial inner membrane-expressed gene *oxa1l* could also affect the biological function of the protein.

**Table 1:** Editing sites with more than 0.15 variation of editing level in genes associated with deafness and ototoxicity.

**ChromeChr**	**Position**	**Variation type**	**Editing in control**	**Editing in gentamycin**	**Editing in neomycin**	**Region**	**Alteration**	**Gene name**	**Gene function**
chr10	2974831	A->G	38.9%	66.4%	67.6%	3′UTR	N/A	*marveld2a*	Non-syndromic hearing loss
chr24	40729176	A->G	66.7%	90.1%	97.9%	3′UTR	N/A	*tbc1d24*	Non-syndromic hearing loss
chr24	40729155	A->G	66.7%	4.5%	45.1%	3′UTR	N/A	*tbc1d24*	Non-syndromic hearing loss
chr21	9763713	A->G	67.5%	42.2%	50.6%	CDS	I/V	*bdp1 (2 of 2)*	Non-syndromic hearing loss
chr21	9763719	A->G	68.3%	44.6%	50.0%	CDS	M/V	*bdp1 (2 of 2)*	Non-syndromic hearing loss
chr12	5897211	A->G	39.3%	60.8%	62.1%	3′UTR	N/A	*plekhm1*	Osteopetrosis
chr12	5897300	A->G	32.8%	59.8%	59.6%	3′UTR	N/A	*plekhm1*	Osteopetrosis
chr12	5897334	A->G	58.3%	79.4%	100.0%	3′UTR	N/A	*plekhm1*	Osteopetrosis
chr12	5896131	A->G	46.0%	25.0%	8.3%	3′UTR	N/A	*plekhm1*	Osteopetrosis
chr12	5897541	A->G	65.1%	39.3%	30.5%	3′UTR	N/A	*plekhm1*	Osteopetrosis
chr20	32527617	A->G	56.1%	4.2%	10.7%	3′UTR	N/A	*ostm1*	Osteopetrosis
chr1	27553904	A->G	45%	19.2%	17.7%	3′UTR	N/A	*ednrb1a*	Waardenburg IV syndrome
chr10	3459366	C->T	88.8%	51.1%	64.1%	3′UTR	N/A	*ptpn11a*	Leopard I syndrome
chr13	8739101	G->A	30.6%	8.4%	15.5%	CDS	R/H	*alms1*	Alstrom Syndrome
chr13	31129396	G->A	28.6%	10.9%	10.0%	CDS	K/K	*ercc6*	Cockayne type B syndrome
chr13	46955730	C->T	77.8%	52.4%	60.6%	Intron	N/A	*fgfr2*	Crouzon & Apert syndromes
chr8	53710690	A->G	61.0%	44.5%	45.5%	3′UTR	N/A	*fgfr1a*	Pfeiffer syndrome
chr8	53711300	A->G	69.4%	28.8%	45.7%	3′UTR	N/A	*fgfr1a*	Pfeiffer syndrome
chr8	53711318	A->G	31.1%	12.2%	9.4%	3′UTR	N/A	*fgfr1a*	Pfeiffer syndrome
chr8	53711323	A->G	30.6%	4.2%	3.1%	3′UTR	N/A	*fgfr1a*	Pfeiffer syndrome
chr12	2152293	A->T	71.7%	28.6%	53.1%	Intron	N/A	*sox9a*	Ear development
chr12	2152698	G->A	45.6%	15.4%	22.0%	Intron	N/A	*sox9a*	Ear development
chr8	17101817	A->G	91.7%	64.3%	69.4%	3′UTR	N/A	*calrl2*	AG binding
chr8	17101934	A->G	74.1%	46.7%	37.7%	3′UTR	N/A	*calrl2*	AG binding

**Table 2:** Missense editing variants with more than 0.15 variation of editing level.

**Chrome Chr**	**Position**	**Variation type**	**Editing in control**	**Editing in gentamycin**	**Editing in neomycin**	**Alteration**	**Gene name**
chr7	20754724	A->G	8.7%	25.7%	29.8%	K/R	*oxa1l*
chr21	7195948	A->G	64.6%	46.4%	47.6%	M/V	*f2rl1.2*
chr21	7196037	A->G	75.6%	52.5%	42.0%	H/R	*f2rl1.2*
chr21	7196041	A->G	74.1%	52.5%	39.1%	N/D	*f2rl1.2*
chr7	53947931	A->G	55.9%	33.3%	20.5%	K/E	*cgnl1*
chr7	53948123	A->G	54.1%	35.0%	30.9%	S/G	*cgnl1*
chr25	32457360	T->C	59.8%	43.0%	32.6%	C/R	*rassf8l*
chr21	9413646	C->T	56.1%	25.0%	30.2%	T/I	*alpk2*
chr6	29017626	C->T	38.5%	22.9%	16.1%	A/T	*bivm*
chr15	5724304	C->T	53.1%	32.1%	34.5%	R/Q	*brwd1*
chr23	17169862	C->T	49.7%	25.9%	13.6%	S/L	*dnmt3*
chr25	29250985	C->T	51.0%	34.7%	23.0%	Q/Stop	*ptprz1a*
chr11	38600377	C->T	57.3%	32.7%	23.0%	T/I	*zgc:113019*
chr16	18334447	G->A	7.4%	26.2%	31.8%	A/T	*setd2*
chr19	539349	G->A	50.3%	34.9%	30.8%	E/K	*akap9*
chr2	49607422	G->A	38.9%	7.4%	14.3%	G/S	*abhd17aa*
chr21	9404526	G->A	57.9%	25.0%	23.3%	G/D	*alpk2*

### Characteristics of RNA sequence context under the AG treatment

**Figure 3:**
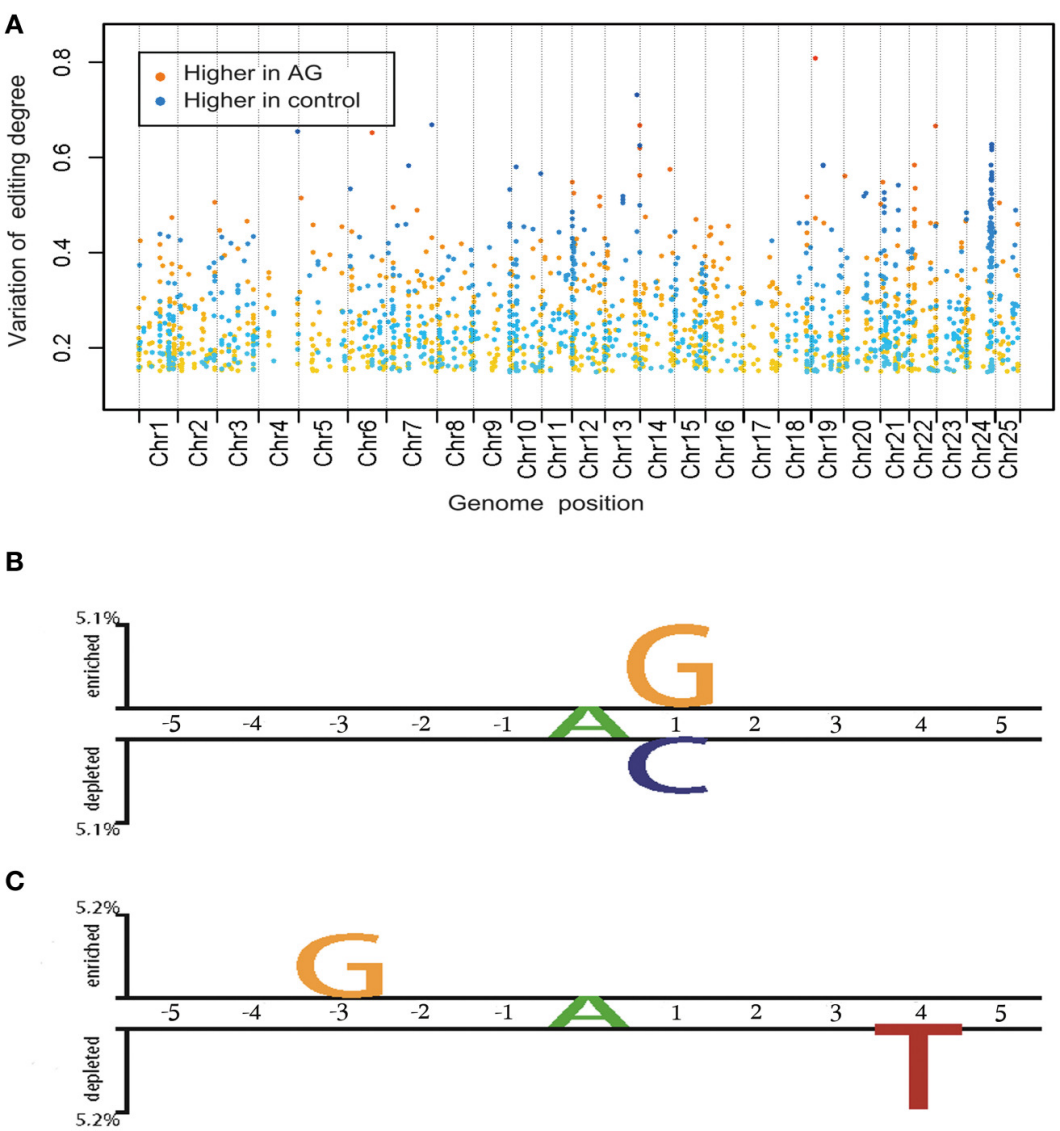
Global biological properties of editome in control and drug treated samples. **(A)** Distribution and fluctuation of whole editome under AG treatment. Each spot represents one editing site, of which the X-axis shows the location on chromosome and Y-axis shows the change of editing degree. Spots are highlighted in colors as follows: from yellow to red, editing degree higher in AG treatment; from green to blue, editing degree higher in wide type, the degree of color deepening was related to the change of editing degree. **(B)** Two sample logo of the differences between editing sites in high variation and low variation groups under drug treatment for the *p*-value threshold of 0.05. **(C)** Two sample logo of the differences between editing sites with higher editing degree in drug treated orwith higher editing degree in control groups for the *p*-value threshold of 0.05.

We wanted to investigate whether there were sequence characteristics underlying the AG targeting of RNA. Therefore, we compared the flanking sequences of editing sites with editing variations >15% of control and AG-treated samples (high variation group) with those that had <5% variation in the degree of editing (low variation group; Figure 3B). We found that sites in the high variation group were flanked by G-enriched sequences and exhibited a depletion of C nucleotides downstream of the edited site. This agrees with previous findings that the C nucleotide is underrepresented at the +1 position of editing sites in zebrafish, while G is overrepresented at the +1 position of editing sites in humans and mice (Shamay-Ramot et al., 2015). This is also in agreement with the 3′ nearest neighbor preferences of the ADAR2 enzymes, with G being preferred (Eggington et al., 2011). Additionally, the presence of a T nucleotide four bases downstream of the edited site was characteristic of sites with down-regulated degrees of editing (more than 15%) in AG-treated samples. Enrichment of G nucleotides three bases upstream of the edited site was characteristics of sites with up-regulated degrees of editing (more than 15%) in AG-treated samples (Figure 3C). Nucleotide preferences in the flanking sequences of RNA editing sites indicate that the binding of AG is facilitated by specific sequence and structural features.

### Comparison of gene expression in AG-treated and control samples

We measured variations in gene expression between the AG-treated and control 2 dpf embryo samples. RNA of all samples were isolated from whole embryos. The expression of 988 genes differed more than 1.5-fold between both neomycin and gentamycin-treated samples and normal control samples. Of these genes, 257 genes were lateral line-enriched genes identified in previous studies (Jiang et al., 2014; Steiner et al., 2014). Among the 722 genes with down-regulated expression in the two AG-treated samples (Supplementary Table 7), 113 genes were also down-regulated more than 1.5-fold in the expression analysis of lateral line cells at 1 h after neomycin-induced hair cell death, including *her6, etv4*, and *tcf7l1a* (Jiang et al., 2014). Twenty-seven of the down-regulated genes, including *ptprq, ush1g, climp-63*, and genes involved in the Wnt, Notch, and FGF signaling pathways, play important roles in ear development or ototoxicity (Karasawa et al., 2010; Stamatiou and Stankovic, 2013; Jiang et al., 2014; Table 3). There were 266 genes with up-regulated expression in AG-treated samples, among which 47 genes were up-regulated more than 1.5-fold in the lateral line cells at 1 h after neomycin-induced hair cell death (Jiang et al., 2014). Pathway analysis showed that down-regulated genes were significantly enriched (*p* < 0.05) in cytoskeletal regulation by Rho GTPase and the thyrotropin-releasing hormone receptor, cadherin, and Wnt signaling pathways (Supplementary Table 8). Up-regulated genes were significantly enriched (*p* < 0.05) in DNA replication and in the apoptosis signaling pathway (Supplementary Table 9).

**Table 3:** Deafness and ototoxicity genes were down-regulated more than 1.5 fold change in AG treated Zebrafish.

**Gene name**	**Control (FPKM)**	**Gentamycin (FPKM)**	**Log (fold)**	**Neomycin (FPKM)**	**Log (fold)**	**Gene related phenotype or function**
*ush1g*	0.44	0.10	−2.10	0.24	−0.86	Syndromic hearing loss
*pcdh15b*	1.51	0.51	−1.58	0.39	−1.96	Non-syndromic hearing loss
*ptprq*	0.49	0.07	−2.78	0.12	−2.02	Non-syndromic hearing loss
*col1a1a*	1001.63	626.27	−0.68	466.45	−1.10	Osteogenesis
*twist1a*	40.15	24.23	−0.73	24.84	−0.69	Syndromic hearing loss
*diabloa*	17.56	9.13	−0.94	8.42	−1.06	Non-syndromic hearing loss
*actb2*	4882.95	2675.42	−0.87	2703.10	−0.85	Deafness (animal experiment)
*tyr*	55.70	33.88	−0.72	22.99	−1.28	Syndromic hearing loss
*lrtomt*	0.63	0.31	−1.01	0.36	−0.81	Non-syndromic hearing loss
*fetub*	1040.03	650.12	−0.68	369.93	−1.49	Deafness (animal experiment)
*plekhm1*	4.75	3.12	−0.60	2.98	−0.67	Osteopetrosis
*fgf10b*	0.44	0.18	−1.30	0.22	−1.01	Syndromic hearing loss
*edn3*	1.84	0.02	−6.53	0.97	−0.93	Syndromic hearing loss
*kctd1*	2.32	0.93	−1.32	1.46	−0.67	Deafness (animal experiment)
*apoa1b*	5865.83	3853.52	−0.61	3544.56	−0.73	Deafness (animal experiment)
*cldn2*	1.05	0.58	−0.84	0.57	−0.88	Non syndromic hearing loss
*dachc*	6.26	4.07	−0.62	3.68	−0.77	Ear development
*fgf5*	0.74	0.27	−1.46	0.22	−1.76	Ear development
*fgf20b*	1.21	0.68	−0.84	0.66	−0.87	Ear development
*etv4*	25.05	14.80	−0.76	12.94	−0.95	Ear development
*snai1b*	4.79	2.98	−0.69	2.95	−0.70	Ear development
*her6*	60.58	37.20	−0.70	34.28	−0.82	Notch signaling pathway
*her12*	116.63	62.82	−0.89	65.52	−0.83	Notch signaling pathway
*tcf7l1a*	37.23	24.73	−0.59	18.14	−1.04	Wnt signaling pathway
*jun*	97.64	60.41	−0.69	36.52	−1.42	Wnt signaling pathway
*sfrp1b*	19.61	9.56	−1.04	7.53	−1.38	Wnt signaling pathway
*climp-63*	143.45	86.49	−0.73	72.02	−0.99	AG binding

### Relationship between editing and gene expression

**Figure 4:**
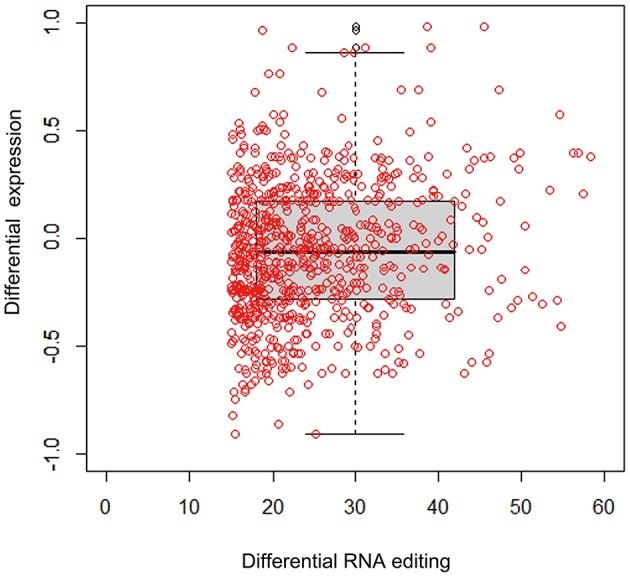
Relationship between editing level and expression change. Correlation between editing level and mRNA level changes of associated genes between gentamycin treated and control groups (Pearson's correlation test: *r* = 0.10, 95% CI: 0.06–0.20, *p* = 0.0088). Each red dot stood for an editing site with a cutoff of 15% editing level change.

The RNA-seq data allowed us to assess the connection between RNA editing and gene expression in the experimental samples. Increases in the level of RNA editing were weakly, but significantly, correlated with increases in gene expression in the gentamycin treatment group (Pearson's correlation test: *r* = 0.10, 95% CI: 0.06–0.20, *p* = 0.0088; Figure 4). Moreover, 160 editing sites were located in 19 genes with more than a 1.5-fold difference in expression between AG-treated and control samples. The degree of editing changed more than 15% in 21 of these editing sites, and all but two were located in non-coding regions (Table 4). Among these 21 editing sites, three were predicted by MIRANDA to alter or create miRNA binding sites and possibly affect the expression of *shmt2, si:ch211-241b2.1*, and *plekhm1* (Table 5). Under AG treatment, the expression levels of *shmt2* and *plekhm1* were down-regulated more than 1.5-fold with the up-regulated editing of these two miRNA binding sites. *shmt2* (serine hydroxymethyltransferase 2) is mainly distributed in the mitochondria and plays an important role in mitochondrial DNA synthesis and glycine production, while *plekhm1* is associated with syndromic hearing loss (Van Wesenbeeck et al., 2007; Anderson and Stover, 2009).

**Table 4:** Genes changed more than 1.5 fold change in expression and more than 15% in editing degree.

**Genome Chr**	**Position**	**Variation Type**	**Editing in Control**	**Editing in Gentamycin**	**Editing in Neomycin**	**Region/Alteration**	**Gene name**	**Expression change**
chr1	37502	A->G	19.4%	43.7%	37.0%	3′UTR	*f10*	Down-regulated
chr12	5897211	A->G	39.3%	60.8%	62.1%	3′UTR	*plekhm1*	Down-regulated
chr12	5897300	A->G	32.8%	59.8%	59.6%	3′UTR	*plekhm1*	Down-regulated
chr12	5897334	A->G	58.3%	79.4%	100.0%	3′UTR	*plekhm1*	Down-regulated
chr19	46815419	A->G	10.0%	28.6%	32.1%	3′UTR	*cu694368.1*	Down-regulated
chr3	55688757	A->G	16.7%	32.2%	32.3%	Intron	*ubald1b*	Down-regulated
chr6	59149763	A->G	5.8%	25.5%	40.5%	3′UTR	*shmt2*	Down-regulated
chr7	20754724	A->G	8.7%	29.8%	25.7%	CDS	*oxa1l*	Down-regulated
chr16	35895953	A->G	36.1%	81.6%	71.9%	Intron	*si:dkey-248g17.3*	Up-regulated
chr12	5896131	A->G	46.0%	25.0%	8.3%	3′UTR	*plekhm1*	Down-regulated
chr12	5897541	A->G	65.1%	39.3%	30.5%	3′UTR	*plekhm1*	Down-regulated
chr12	26797012	A->G	91.7%	72.0%	76.4%	Intron	*mta3*	Down-regulated
chr12	26797182	A->G	28.5%	12.9%	10.6%	Intron	*mta3*	Down-regulated
chr12	26797214	A->G	70.0%	53.3%	37.5%	Intron	*mta3*	Down-regulated
chr12	26797227	A->G	32.1%	7.7%	14.1%	Intron	*mta3*	Down-regulated
chr19	46815518	A->G	59.4%	18.8%	43.8%	3′UTR	*cu694368.1*	Down-regulated
chr20	36940734	C->T	73.2%	39.5%	45.1%	3′UTR	*cited2*	Down-regulated
chr3	55687706	A->G	51.3%	30.7%	36.2%	Intron	*ubald1b*	Down-regulated
chr3	55687778	A->G	33.2%	16.8%	10.8%	Intron	*ubald1b*	Down-regulated
chr15	826092	C->A	63.3%	22.0%	18.8%	CDS	*si:dkey-7i4.7*	Up-regulated
chr21	2596180	G->A	54.3%	14.3%	26.0%	3′UTR	*si:ch211-241b2.1*	Up-regulated

**Table 5:** Genes with editing sites in potential miRNA binding elements changed more than 15% in editing degree and 1.5-fold in expression.

**Genome**		**Variation Type**	**Editing degree in different treatment**			**miRNA binding in editing sites**		**Gene name**	**Expression Change ≥1.5 fold change**
**Chr**	**Position**		**Control**	**Genta**	**Neo**	**Before editing**	**After editing**		
chr12	5897334	A->G	58.3%	79.4%	100.0%	N/A	dre-miR-726: G|C	*plekhm1*	Down-regulated
chr21	2596180	G->A	54.3%	14.3%	26.0%	dre-miR-735-3p: G|C	dre-miR-26a-2-3p: A|U	*si:ch211-241b2.1*	Up-regulated
chr6	59149763	A->G	5.8%	25.5%	40.5%	N/A	dre-miR-727-5p: G|C	*shmt2*	Down-regulated

## Discussion

RNA editing is an essential gene regulatory mechanism and is quite prevalent throughout the transcriptome. Deficiencies in ADAR can lead to lethality or severe defects of the nervous system in flies, zebrafish, and mice (Palladino et al., 2000; Horsch et al., 2011; Li et al., 2014). In humans, the deregulation of RNA editing is associated with some types of cancer and several neurological diseases (Eran et al., 2013; Gallo et al., 2017). The dynamic regulation of RNA editing in the human brain was found to be associated with neuronal maturation, while hyper-editing was selectively perturbed in spinal cord injury and glioblastoma (Hwang et al., 2016). There is increasing evidence to support that RNA editing is one of molecular mechanisms connecting environmental stimuli and phenotypic or behavioral output.

With the aid of high-throughput RNA sequencing technologies, more and more RNA editing sites have been identified in humans (*Homo sapiens*; Ramaswami et al., 2013), mice (*Mus musculus*; Danecek et al.), and flies (*Drosophila melanogaster*; St Laurent et al., 2013). Previous study by Ramaswami et al. identified 370,623 RNA editing sites in the human transcriptome, while a study by Danecek et al. found 7,389 editing sites in the mouse brain (Danecek et al., 2012; Ramaswami et al., 2013). Additionally, among 1,745 genes contained editing sites in mice, 1,206 genes were also reported to contain editing sites in humans (Gu et al., 2012). A recent study identified ~350,000 DNA–RNA mismatches using a dataset that contained 17 zebrafish samples covering eight different developmental stages (Shamay-Ramot et al., 2015). RNA-seq data have been analyzed using a bioinformatics pipeline masking adenosine (A) sites with guanine (G) to find extensive hyperedited RNA sites (Porath et al., 2014). Here, 6,850 mRNA editing events in embryonic zebrafish at 2 dpf were identified by next-generation sequencing technologies and the recently published SPRINT method (Zhang et al., 2017). We found that 74% of all editing sites were located in the 3′ UTR regions of genes. It will be an exciting challenge to uncover the roles of these editing sites in biology and disease in future studies.

AG have been used to treat serious or recalcitrant gram-negative infections for more than 70 years (Schatz and Waksman, 1944). However, the clinical utility of these antibiotics is limited because they induce nephrotoxicity and ototoxicity (Rizzi and Hirose, 2007). AG-induced ototoxicity is associated with several human mitochondria variants. Individuals who carry 1555A-G or 1494C-T mutations in the mitochondrial 12S rRNA gene are susceptible to AG-induced ototoxicity, as these transitions make human mitochondrial ribosomes more “bacteria-like” for AG binding (Guan, 2011). Moreover, people who do not carry mutations in the 12S rRNA gene also suffer from AG-induced hearing loss, indicating a more complex mechanism of AG-induced ototoxicity.

After systemic administration of AG, damage occurs to the sensory hair cells, which is innervated by neurons of the statoacoustic ganglion (SAG; Sone et al., 1998). The inhibition of mitochondrial protein synthesis and induction of mitochondrial dysfunction may be ways that AG exert toxicity when accumulating in hair cells (Dehne et al., 2002). AG also initiate multiple pathways, including the formation of reactive oxygen species (Rybak and Ramkumar, 2007), the activation of the c-Jun N-terminal kinase pathway, and caspase-dependent or -independent signals (Jiang et al., 2006), leading to necrotic or apoptotic cell death.

Clinically, AG-induced hearing loss is one of the main causes of drug-induced deafness in children which could be partly due to newborns and infants are more susceptible to drug-induced ototoxicity than adults (Scaglione et al., 1995; Li et al., 2005). Embryonic zebrafish as a model to carry out the ototoxic effects of AG may effectively increase awareness of AG-ototoxic pathogenic mechanism in young children.

The present study assessed the dynamic variation of the editome after exposure to AG. The high variations of editing in *plekhm1, sox9a, fgfr1a*, and *fgfr2* observed under AG treatment indicated potential effects of AG on inner ear development. Each of these genes is involved in some aspect of inner ear development. *plekhm1* plays an important role in the development and formation of the otic capsule (Van Wesenbeeck et al., 2007). *sox9a* is a target of the FGF3 and FGF8 signaling pathways (Nicolson, 2005). *fgfr1a* is prominently expressed in neuromasts of the posterior lateral line and is important in the generation of the precursor pool that grows into the auditory sensory epithelium (Pirvola et al., 2002). *fgfr2*, a cognate receptor of *fgf3* and *fgf10*, is expressed in the otocyst nonsensory epithelium and regulates inner ear morphogenesis (Pirvola et al., 2000). Notably, *calrl2*, which encodes a protein that binds to gentamicin to reduce drug-induced ototoxicity, contains three editing sites in the 3′ UTR, among which the editing level of two sites was largely down-regulated under AG treatment (Karasawa et al., 2011). Thus, for the first time, we have demonstrated that AG have a significant effect on the biological properties of mRNA editing sites. This finding provides new insight into the mechanisms of AG-induced ototoxicity. The mechanism of AG induced editing variation may be that AG bind to specific internal loop motif to affect the formation of dsRNA that are edited by ADAR (Lehmann and Bass, 1999; Disney et al., 2008), AG may affect the affinity of ADAR and substrate RNA, and then result in the change of editing efficiency.

Expression profile analysis also showed that the expression of some members, or targets, of the FGF signaling pathway were down-regulated more than 1.5-fold under AG treatment (Raible and Brand, 2001). The FGF signaling pathway regulates the developmental processes of the inner ear, including the ontogeny of the SAG and hair cells in zebrafish (Wang et al., 2015). *fgf5*, expressed in mature SAG neurons (Vemaraju et al., 2012) and some other cranial ganglia from 24 to 48 hpf, was the most down-regulated gene in this pathway. *fgf5* plays a major role in slowing the rate of maturation of new SAG neurons and in expanding the size of the progenitor cell pool for future use. Taken together, the depletion of *fgf5* after AG treatment would be expected to promote the maturation of neurons and advance the deletion of progenitors, leading to an overall SAG deficiency.

Moreover, the expression of some hearing loss-related genes was down regulated after treatment with AG, with *ptprq* expression decreasing the most. *Ptprq* encodes the cytoplasmic protein tyrosine phosphatase receptor Q, which has activity against phosphatidylinositol phosphates (Wright et al., 1998). Protein tyrosine phosphatase receptor Q regulates the local phosphoinositide phospholipid content of the hair cell apical membrane (Oganesian et al., 2003) and participates in the normal maturation of developing cochlear hair bundles (Goodyear et al., 2003). AG bind to free phosphoinositides, which regulate KCNQ4 channel activity, leading to the inhibition of the potassium efflux necessary for the survival and function of cochlear sensory hair cells (Leitner et al., 2011). The down-regulation of phosphoinositide in the specific sensory tissue by *ptprq* is likely to be responsible for the selective susceptibility of inner ear hair cells to AG.

Meanwhile, genes with more than 1.5-fold down-regulated expression in AG-treated samples were enriched in the Wnt and cadherin signaling pathways. Wnt signaling is required for proliferation in developing neuromasts, and there are intriguing connections between the Wnt and cadherin signaling pathways in AG-induced ototoxicity (Nelson and Nusse, 2004; Head et al., 2013). As one of genes in the Wnt signaling pathway, *tcf7l1a* is a conserved transcription factor whose function as a transcriptional repressor is necessary for early nervous system development in zebrafish (Dorsky et al., 2003). The down-regulated expression of *tcf7l1a* was also reported after treatment with neomycin in a previous study (Jiang et al., 2014).

In addition, two mitochondria-related genes, *oxa1l* and *shmt2*, showed changes in both editing degree and expression after exposure to AG. The *oxa1l* gene encodes an evolutionarily conserved mitochondrial inner membrane protein, the C-terminal tail of which binds mitochondrial ribosomes, coordinating the synthesis and membrane insertion of the nascent chains into the membrane (Haque et al., 2010). Knocking out this gene in HEK293 cells leads to a significant decrease in the steady-state level and activity of mitochondrial F (1) F (o)-ATP synthase (Stiburek et al., 2007). Moreover, the editing sites of *shmt2* were all located in the 3′ UTR region, and the editing degree variation in one of these sites may influence its binding with an miRNA under AG treatment. *shmt2* plays an important role in the synthesis of mitochondrial glycine for mitochondrial DNA generation. The down-regulation of *shmt2* expression in HeLa cells affects the integrity of the mitochondrial DNA content and in elderly human fibroblast lines regulates glycine production in the mitochondria, resulting in respiration defects (Anderson and Stover, 2009; Hashizume et al., 2015).

## Conclusion

Taken together, AG may widely influence the editome and expression profiles of genes, and these changes are potentially correlated with AG-induced ototoxicity. These results provide new insight into the ototoxic mechanism of AG. However, the interactions between AG, RNA editing, and hearing loss involve complex processes, and additional studies are required to fully elucidate the mechanisms involved. A better understanding of post-transcriptional editing events may not only help to improve our understanding of the pathogenesis of AG ototoxicity but also lead to the design of novel strategies for disease treatment.

## Author contributions

SY, YL, LH, and QX: designed and supervised the project; SY, HZ, MJ, and YS: performed the experiments; SY, YL, WT, and LW: analyzed the data; SY, YL and XZ: wrote the manuscript. All authors read and approved the final manuscript. All authors contributed to the interpretation of data, and revised the manuscript.

### Conflict of interest statement

The authors declare that the research was conducted in the absence of any commercial or financial relationships that could be construed as a potential conflict of interest.

## Supplementary material

Supplementary Image 1 Validation of inferred editing sites from RNA-Seq by Sanger sequencing. Sequencing chromatogram traces from gene loci *SP4* and *Si: ch211-114n24.7*are shown. The editing positions (located in the 3′UTR of *SP4* and 5′ UTR of *Si: ch211-114n24.7*) are highlighted by yellow shading. Note the clustering of editing sites in the *SP4* transcript. Top trace is genomic DNA (gDNA), bottom trace cDNA.
