# Supplementary figures and images for: Dynamic Editome of Zebrafish under Aminoglycosides Treatment and Its Potential Involvement in Ototoxicity
Source: Front Pharmacol. 2017 Nov 22;8:854. doi: 10.3389/fphar.2017.00854 (PMC5702851; doi:10.3389/fphar.2017.00854)

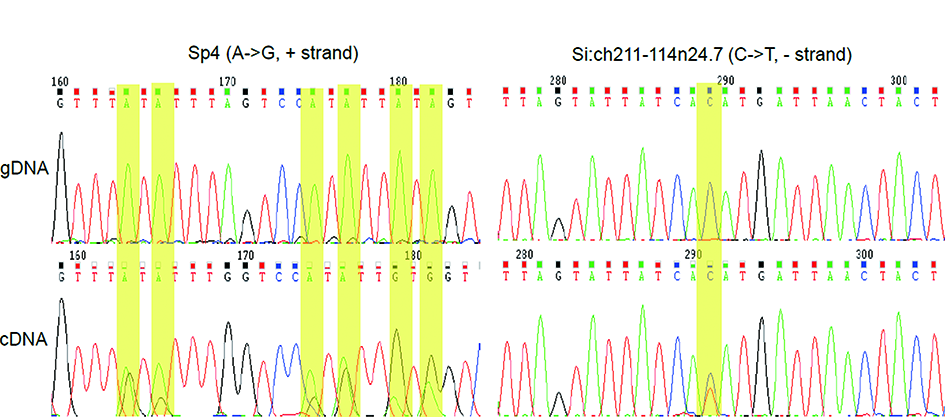

Supplement: Supplementary Image 1 — Validation of inferred editing sites from RNA-Seq by Sanger sequencing. Sequencing chromatogram traces from gene loci SP4 and Si: ch211-114n24.7are shown. The editing positions (located in the 3′UTR of SP4 and 5′ UTR of Si: ch211-114n24.7) are highlighted by yellow shading. Note the clustering of editing sites in the SP4 transcript. Top trace is genomic DNA (gDNA), bottom trace cDNA. [file Image1.tif]
