# Supplementary material for: Dynamic Editome of Zebrafish under Aminoglycosides Treatment and Its Potential Involvement in Ototoxicity
Source: Front Pharmacol. 2017 Nov 22;8:854. doi: 10.3389/fphar.2017.00854 (PMC5702851; doi:10.3389/fphar.2017.00854)
Supplement: Supplementary file 5 [file Table4.DOCX]

| **Pathway** | **p-Value** |
| --- | --- |
|  |  |
| **Pyrimidine Metabolism** | 0.026 |
| **Axon guidance mediated by semaphorins** | 0.001 |
| **Alzheimer disease-presenilin pathway** | 0.003 |
| **Angiogenesis** | **0.019** |
| **FGF signaling pathway** | 0.004 |
| **Cytoskeletal regulation by Rho GTPase** | 0.017 |
| **Ras Pathway** | 0.038 |
| **Huntington disease** | 0.008 |

**Supplementary Table S4. Significantly overrepresented pathways of genes with mRNA editing sites in 2 dpf zebrafish.**

| **Pathway** | **p-Value** |
| --- | --- |
|  |  |
| **Pyrimidine Metabolism** | 0.026 |
| **Axon guidance mediated by semaphorins** | 0.001 |
| **Alzheimer disease-presenilin pathway** | 0.003 |

| **Pathway** | **p-Value** |
| --- | --- |
|  |  |
