# Supplementary material for: Dynamic Editome of Zebrafish under Aminoglycosides Treatment and Its Potential Involvement in Ototoxicity
Source: Front Pharmacol. 2017 Nov 22;8:854. doi: 10.3389/fphar.2017.00854 (PMC5702851; doi:10.3389/fphar.2017.00854)
Supplement: Supplementary file 7 [file Table6.DOCX]

**Supplementary Table S6. Significantly Overrepresented Pathways of 333 genes in the high variation group**

| **Pathway** | **p-Value** |
| --- | --- |
| **Proline biosynthesis** | 0.032 |
| **Tetrahydrofolate biosynthesis** | 0.043 |
| **Axon guidance mediated by netrin** | 0.018 |
| **FGF signaling pathway** | 0.006 |
| **Ras Pathway** | 0.023 |
