# Supplementary material for: Dynamic Editome of Zebrafish under Aminoglycosides Treatment and Its Potential Involvement in Ototoxicity
Source: Front Pharmacol. 2017 Nov 22;8:854. doi: 10.3389/fphar.2017.00854 (PMC5702851; doi:10.3389/fphar.2017.00854)
Supplement: Supplementary file 9 [file Table8.DOCX]

**Supplementary Table S8. Significantly overrepresented pathway of 722 genes with down-regulated expression in the two AG-treated samples**

| **Pathway** | **p-Value** |
| --- | --- |
| **Cytoskeletal regulation by Rho GTPase** | 0.012 |
| **Thyrotropin-releasing hormone receptor signaling pathway** | 0.041 |
| **Cadherin signaling pathway** | 0.034 |
| **Wnt signaling pathway** | 0.018 |
