# Supplementary material for: Dynamic Editome of Zebrafish under Aminoglycosides Treatment and Its Potential Involvement in Ototoxicity
Source: Front Pharmacol. 2017 Nov 22;8:854. doi: 10.3389/fphar.2017.00854 (PMC5702851; doi:10.3389/fphar.2017.00854)
Supplement: Supplementary file 10 [file Table9.DOCX]

**Supplementary Table S9. Significantly overrepresented pathway of 226 genes with up-regulated expression in the two AG-treated samples**

| **Pathway** | **p-Value** |
| --- | --- |
| **DNA replication** | 4.4×10^-12^ |
| **Apoptosis signaling pathway** | 0.033 |
